# Histopathological Analysis of Vacuum-Assisted Breast Biopsy in Relation to Microcalcification Findings on Mammography: A Pictorial Review

**DOI:** 10.3390/biomedicines13030737

**Published:** 2025-03-18

**Authors:** Jana Bebek, Nikolina Novak, Marina Dasović, Eugen Divjak, Čedna Tomasović-Lončarić, Boris Brkljačić, Gordana Ivanac

**Affiliations:** 1 Department of Diagnostic and Interventional Radiology, University Hospital Dubrava, 10000 Zagreb, Croatiagordana.ivanac@mef.hr (G.I.); 2 Department of Pathology and Cytology, University Hospital Dubrava, 10000 Zagreb, Croatia; 3 School of Medicine, Catholic University of Croatia, 10000 Zagreb, Croatia; 4 School of Medicine, University of Zagreb, 10000 Zagreb, Croatia

**Keywords:** breast cancer, breast oncology, breast biopsy, microcalcifications, histopathological analysis

## Abstract

Mammography is an essential tool in breast screening, often revealing lesions that appear as microcalcifications with or without an associated mass. Decisions about biopsy requirements are guided by the BI-RADS system, aiming to confirm the histopathology of suspicious lesions while avoiding unnecessary procedures. A vacuum-assisted breast biopsy (VABB) is a minimally invasive procedure for diagnosing breast abnormalities. Precise lesion targeting is ensured under stereotactic guidance, reducing the need for repeated procedures. Compared to traditional core needle biopsy (CNB) and fine-needle aspiration cytology (FNAC), it differs in using vacuum assistance to gather more tissue volume, increasing diagnostic accuracy and reducing the likelihood of histological underestimation. This is particularly crucial in cases where microcalcifications are the primary finding, as they are often the earliest signs of ductal carcinoma in situ (DCIS). Managing such findings requires precise diagnostic tools to differentiate benign from malignant lesions without subjecting patients to unnecessary surgical interventions. Building on several years of experience in our department, we have assembled a selection of ten interesting cases encountered in our clinical practice. Each case is documented with paired mammographic images and their corresponding image of histopathological findings, offering a comprehensive view of the diagnostic journey. These cases were selected for their educational value, highlighting the integration of imaging modalities, histopathological evaluation, and clinical decision-making. All cases underwent an extensive diagnostic workup at our facility. This compilation aims to provide valuable insights for both clinicians and researchers, offering a deeper understanding of advanced diagnostic techniques and their role in improving patient outcomes.

## 1. Introduction

Breast cancer in women is the second most common cause of cancer-related deaths (11.6%) [1]. Early detection of breast cancer helps lower morbidity and mortality while improving survival rates among patients. Mammography and ultrasound (US) are primary imaging methods used for breast screening, while magnetic resonance imaging (MRI) is typically reserved for screening high-risk individuals. The increasing interest in breast cancer and the awareness of breast cancer screening programs have led to the detection of various lesions. Lesions found through screening are usually non-palpable and often appear as calcifications with or without an associated mass. Managing indeterminate breast calcifications can be challenging, and histopathological confirmation is frequently required [2,3,4]. Breast calcifications are a common finding in mammography, associated with both benign and malignant conditions, and serve as a critical early indicator of breast cancer. Most (75%) ductal carcinoma in situ (DCIS) cases are detected through mammography without a palpable mass, with microcalcifications being the earliest sign [5]. Less common mammographic abnormalities include masses, architectural distortion, and asymmetries. While calcifications are vital for early breast cancer detection, most of them are linked to benign breast lesions. Analyzing the distribution patterns and morphology of breast microcalcifications is essential [6].

## 2. BI-RADS Classification and Management

The Breast Imaging Reporting and Data System (BI-RADS) was introduced by the American College of Radiology (ACR) to standardize mammography reporting and ensure quality control. This system aimed to reduce variability in radiology reports by establishing a structured format, a lexicon for imaging findings, and a risk-based categorization system for lesions, with recommended management steps. Using scientific data, the ACR developed descriptors reliably correlating with the likelihood of benign or malignant disease. BI-RADS also includes risk categories estimating malignancy probabilities, from near-zero to over 95%, which clarifies recommendations and reduces ambiguity for non-radiologists [7,8]. If an examination is incomplete, meaning additional imaging needs to be performed or the current results need to be compared to the previous, the report is marked as BI-RADS 0. Categories BI-RADS 1 and 2 have a 0% likelihood of malignancy and do not require further diagnostic steps. Lesions described as probably benign (BI-RADS 3) do not require cytologic or tissue sampling. However, periodic mammography examination is recommended to detect its possible progression in those cases. That way, malignant lesions can be diagnosed in the early stages while still having a favorable prognosis. Following the BI-RADS classification, a lesion described as a category 3 has less than a 2% likelihood of malignancy [9]. Comparing mammograms with previous imaging is important when considering a probably benign diagnosis. Mammographic findings that are described as likely benign include localized findings that typically appear in a single segment of one breast, with the most common types being non-calcified solid masses, a group of punctate calcifications, and focal asymmetric densities. There are cases in which an exception is made, and a biopsy is performed instead of a follow-up, usually because of the patient’s preference or significant clinical concern [7,10]. Biopsy is recommended for all BI-RADS 4 and 5 lesions. The BI-RADS 4 category is designated for findings that, while not having the classic features of malignancy, are suspicious enough to warrant a biopsy recommendation. There is a broad range of malignancy likelihoods for BI-RADS 4, from 2% to 95%. As a result, most recommendations for breast interventional procedures fall under this category [7]. Studies have shown that BI-RADS 4 lesions reported actual malignancy at approximately 34.8% [11]. BI-RADS 5 is described as highly likely to be malignant. Still, with the widespread adoption of imaging-guided percutaneous biopsy, current oncologic management nearly always involves confirming malignancy through tissue sampling before surgery to guide treatment decisions. BI-RADS 6 category is reserved for already known malignancy. The importance of accurate classification within the BI-RADS system cannot be overstated, as misclassification can lead to either unnecessary biopsies or missed malignancies [7].

## 3. Diagnostic Interventional Procedures

Diagnostic interventional procedures are minimally invasive methods that involve tissue or fluid sampling for histopathological or cytological analysis, offering valuable insights into disease characteristics and optimal treatment plans. These procedures reduce the need for unnecessary surgeries, improve patient outcomes, and enhance care quality because they give precise diagnostic information. Breast biopsy methods differ in the thickness of the needle, type of device, and imaging method used for monitoring. The three most common types of sampling procedures are fine-needle aspiration cytology (FNAC), US-guided core needle biopsy (CNB), and vacuum-assisted breast biopsy (VABB) under stereotactic guidance [12]. MRI-guided biopsy and surgical biopsy are used less frequently. Verification of breast cancer by percutaneous biopsy allows better preoperative planning and lowers the number of breast surgeries [13]. Furthermore, after analyzing a tissue sample, personalized treatment strategies are accessible, including chemotherapy and hormonal therapy provided before surgery, known as neoadjuvant therapy [14]. Surgical excision is indicated only when results from minimally invasive procedures are discordant with imaging findings [15,16]. The recommended biopsy technique for masses is US-guided CNB; for microcalcification foci or architectural disorganization, stereotactic biopsy is advised. For large areas of microcalcifications with an associated US-detected nodule, US-guided CNB is recommended, and for cysts or lymphadenopathies (axillary, supra- or sub-clavicular) FNAC is recommended [17]. When a lesion is clearly visible on US imaging, US-guided biopsy is preferred due to its easy approach, shorter procedure time, and lower cost. If a lesion cannot be clearly identified by the US, other imaging methods, such as mammography or MRI, are used for guidance. Typically, mammography or tomosynthesis is chosen for suspicious calcifications and architectural distortions, while MRI is reserved for lesions visible only on MRI [18].

### 3.1. Fine-Needle Aspiration Cytology (FNAC)

FNAC is a minimally invasive technique that collects cellular samples using a thin needle (21–25 gauge) for cytological analysis, typically under US guidance. Common indications include sampling axillary lymph nodes in suspected breast malignancies, rapid diagnostic needs, and therapeutic draining of symptomatic cysts [17]. FNAC is not a primary diagnostic tool for malignancies due to the small sample size and high rate of inadequate samples. Compared with CNB, FNAC is associated with a higher frequency of insufficient samples [19]. FNAC has limitations in distinguishing atypical ductal hyperplasia (ADH) from DCIS and DCIS for invasive cancer [20]. It is also not reliable for the examination of microcalcifications. FNAC cannot provide detailed information about tumors, such as biomarker status, which is especially important when considering neoadjuvant treatment. For this reason, FNAC is increasingly being replaced by CNB or VABB for diagnosing breast lesions [16].

### 3.2. US-Guided Core Needle Biopsy (CNB)

CNB is the standard technique for diagnosing palpable and non-palpable breast lesions using a 12–16-gauge needle. CNB is preferred over FNAC as it provides tissue samples suitable for histopathological examination, thus enabling more precise diagnosis. The samples are obtained with an automated (spring-loaded) gun, typically under the guidance of the US [14]. US monitoring relies on the ability to locate the targeted lesion that was previously detected by palpation, mammography, or MRI. Unlike FNAC, CNB requires the use of local anesthesia. Tissue cylinders are collected, fixed in formalin, embedded in paraffin, and sectioned for multi-level examination. This provides essential tumor characterization, including invasiveness, grade, hormonal receptors, and genetic markers. Additional immunohistochemical and molecular tests can be performed, which are crucial for the identification of additional prognostic and predictive markers. Furthermore, with the advancement of (neo)adjuvant treatments, analyzing individual tumor samples using immunohistochemistry and molecular testing is becoming more important [21]. In a study by Saha et al., FNAC and CNB were compared, revealing that although their specificity was similar (100%), CNB offered greater sensitivity (88.3% compared to 69%) and accuracy (86% compared to 74%) [20].

### 3.3. Vacuum-Assisted Breast Biopsy (VABB) Under Stereotactic Guidance

Vacuum-assisted breast biopsy (VABB) has emerged as a significant advancement in the minimally invasive diagnostics and management of breast lesions. VABB is essentially a type of CNB as “core” refers to the method of collecting a tissue sample, where a hollow needle removes a small cylinder, or “core”, of tissue for analysis. Both VABB and traditional CNB use a needle to extract core tissue samples, so they share the exact primary mechanism. However, VABB differs in using vacuum assistance to gather more tissue volume. To clarify terminology, the term “VABB” is usually used to specifically denote the vacuum-assisted variant, while “CNB” is often reserved to refer to traditional, non-vacuum core needle biopsies. As noted earlier, VABB is precious for its ability to obtain larger tissue samples compared to CNB due to vacuum-assisted devices, thereby enhancing diagnostic accuracy and reducing the likelihood of histological underestimation [22]. In a study by Zhang et al., the false-negative rate for VABB using a 10-gauge needle was 4.9%, while for CNB with a 14-gauge needle, it was 7.8%, both under US guidance. This indicates that VABB has a lower false-negative rate compared to CNB [23]. Additionally, the advantage of the larger tissue sample is that 92% of benign breast lesions with a size of less than 15 mm could be entirely removed with VABB [24]. Stereotaxy is a technique used to identify the precise location of a targeted lesion. This method involves determining the lesion’s three-dimensional position by analyzing two angled mammographic images and calculating the depth (or Z-axis) using parallax. When possible, stereotactic guidance is favored over grid-based guidance in mammography, as it offers greater accuracy in determining the lesion’s Z position, operates more quickly, and exposes the patient to less radiation. The explained approach is applicable to any lesion that can be detected on mammography [25]. The primary factor behind improved biopsy performance under stereotactic mammography guidance is the detection of microcalcifications. Most DCIS lesions are associated with microcalcifications, and studies suggest that VABB retrieved calcifications more frequently than a CNB (98% versus 83%) [26,27]. Due to the larger diameter of samples (needle size usually used is from 8 to 11 gauge), fewer cores can be taken, reducing the chance of complications. The most common complication is hematoma. However, stereotactic biopsy has its disadvantages. It cannot be performed in some cases due to proximity to the thoracic wall or skin, breast thickness, poor visualization of the lesions, or the patient’s inability to lie in the prone position. A study by Mannu et al. in 2019 has shown that stereotactic biopsy could not be performed in 2.3% of all instances because either the breast tissue was too thin (67%) or the microcalcifications were situated in the far posterior region (33%) [28]. VABB can be performed under other imaging modalities, including US and MRI, making it a practical option for clinicians. VABB under MRI is particularly useful for lesions that are not visible on conventional imaging so one can target and sample accurately [29]. The VABB process starts by taking mammogram images from various angles, which are used to precisely locate the biopsy site. A computer analyzes the breast X-rays and indicates the precise location in the abnormal area where the needle tip should be placed. Most stereotactic systems calculate the three-dimensional coordinates of a breast lesion using two images taken 30° apart. After the breast has been compressed in a mammography machine, a biopsy device is inserted into the breast, and additional images are captured to ensure the device is correctly positioned for sample collection. Several tissue samples are taken, after which the device is removed, leaving a biopsy marker in place. A final mammogram is performed to verify the marker’s location. It is essential to take an additional X-ray of the biopsy samples to confirm proper sampling, as the calcifications in the sample should resemble the targeted ones [17].

## 4. Histopathological Classification

The most commonly used histopathological classification for breast lesions is the B-classification. This system is used to interpret biopsy results and guide patient management. The system classifies lesions ranging from normal findings to confirmed malignancies. The main goals of minimally invasive diagnostics of breast lesions are diagnosing malignancy and distinguishing invasive from in situ carcinoma. It is also important to make definite benign diagnoses, thus reducing the need for surgery. B1 (normal) indicates normal breast tissue components such as lobules, ducts, and stroma. This result suggests no malignancy. B2 (benign) represents definite benign diagnoses such as fibroadenoma or fibrocystic changes. The B3 (uncertain malignant potential) lesions represent a heterogeneous group of breast lesions characterized by several overlapping findings in imaging, but histologically, they are distinct and defined entities. Most common B3 lesions include ADH, flat epithelial atypia (FEA), classical lobular neoplasia (LN), radial scar (RS), papillary lesions (PL) without atypia, and phyllodes tumors (PT) [30]. The decision to perform further examination is usually made by a multidisciplinary breast team in consultation with the patient. This may involve excision or additional biopsy [31]. B4 (suspicious of malignancy) is used for lesions that require further investigation because of the high probability of malignancy. The B5 category is used for a definite diagnosis of malignancy, and it is divided into three subcategories: B5a for in situ lesions, B5b for invasive malignancies, and B5c for malignancies that cannot be clearly classified as in situ or invasive [32].

## 5. A Pictorial Review of Ten Cases

The evaluation of mammographic microcalcifications plays a crucial role in early breast cancer detection, with VABB serving as a promising diagnostic tool. To complement the theoretical part of this paper with practical insights and emphasize the importance of VABB in the diagnostics of suspicious breast lesions, we present a pictorial review featuring ten representative cases from our clinical practice. These cases were carefully selected for their educational value and the complexity of findings, aiming to illustrate the diagnostic process thoroughly. Each case includes mammographic images with precise descriptions of microcalcifications and other radiological features that required further diagnostic investigation. These are accompanied by images of histopathological findings of the tissue obtained by VABB, enabling a complete correlation between radiological and histological findings. This approach highlights the critical diagnostic steps involved in clinical decision-making. We believe these cases will serve as a valuable resource for understanding the importance of precise evaluation of microcalcifications and early detection of malignant lesions.

**Figure 1:**
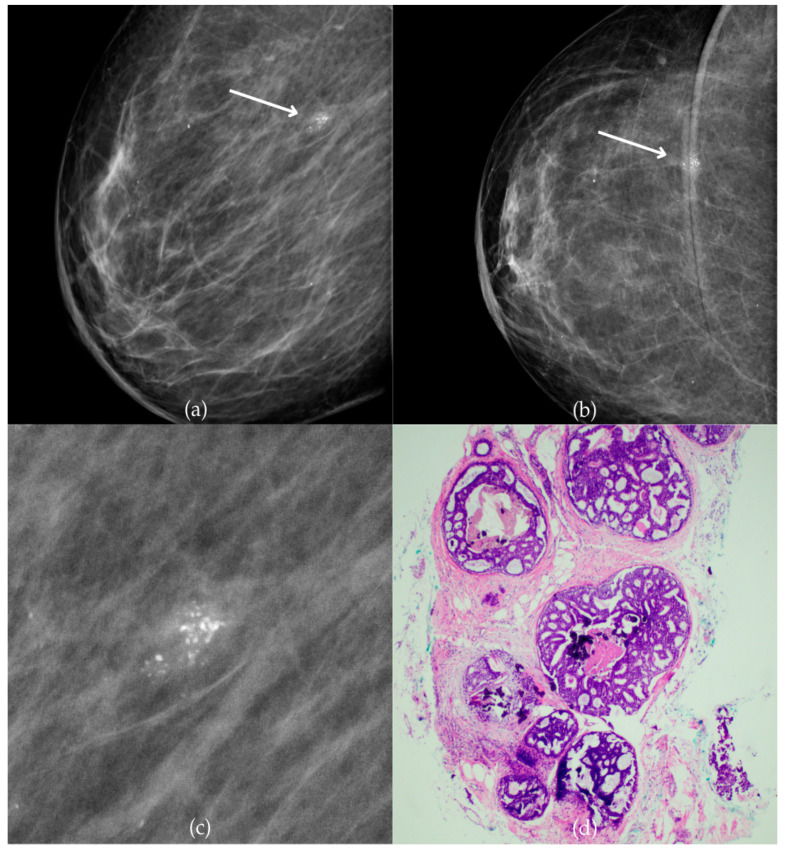
Ductal carcinoma in situ (DCIS). (**a**) Mediolateral oblique (MLO) mammography view; (**b**) craniocaudal (CC) mammography view of the right breast showing grouped coarse heterogeneous and fine pleomorphic calcifications (marked with arrows) in the upper outer quadrant. (**c**) Magnified mammographic image of grouped coarse heterogeneous and fine pleomorphic calcifications. (**d**) The histopathological image of the biopsy sample reveals the characteristic features of DCIS (HE staining; 40× magnification).

Figure 1 presents mammographic and histopathological images of a 74-year-old patient. The mammogram of the right breast shows grouped coarse heterogeneous and fine pleomorphic calcifications in the upper outer quadrant, classified as a BI-RADS 4 finding. Given the suspicious nature of these findings, a biopsy was performed. A tissue analysis confirmed the presence of a DCIS (category B5a). The patient underwent quadrantectomy, and the postoperative pathology confirmed the diagnosis. At the most recent follow-up, conducted one year after the initial diagnosis, the US examination was classified as BI-RADS 2, indicating benign findings.

**Figure 2:**
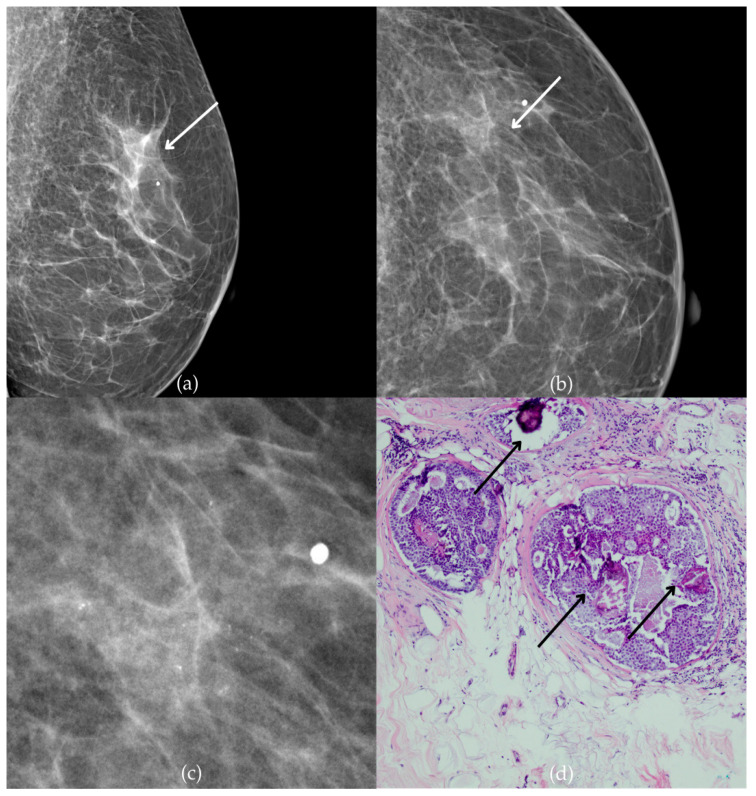
Ductal carcinoma in situ (DCIS). (**a**) Mediolateral oblique (MLO) view; (**b**) craniocaudal (CC) view of the left breast showing architectural distortion with associated grouped amorphous calcifications (marked with arrows) in the upper outer quadrant. (**c**) Magnified mammographic image of grouped amorphous calcifications within architectural distortion. (**d**) The histopathological image of the biopsy sample shows the characteristic features of DCIS with calcifications (marked with arrows) (HE staining; 100× magnification).

In Figure 2, mammographic and histopathological images illustrate the findings in a 51-year-old patient. The mammogram of the patient’s left breast shows architectural distortion with associated grouped amorphous calcifications over the 1 cm area in the upper outer quadrant, corresponding to a BI-RADS 4 finding. Tissue analysis obtained through biopsy confirmed morphological aspects of the DCIS (category B5a). Two years after the initial diagnosis, the most recent US follow-up was classified as BI-RADS 2, confirming benign findings.

**Figure 3:**
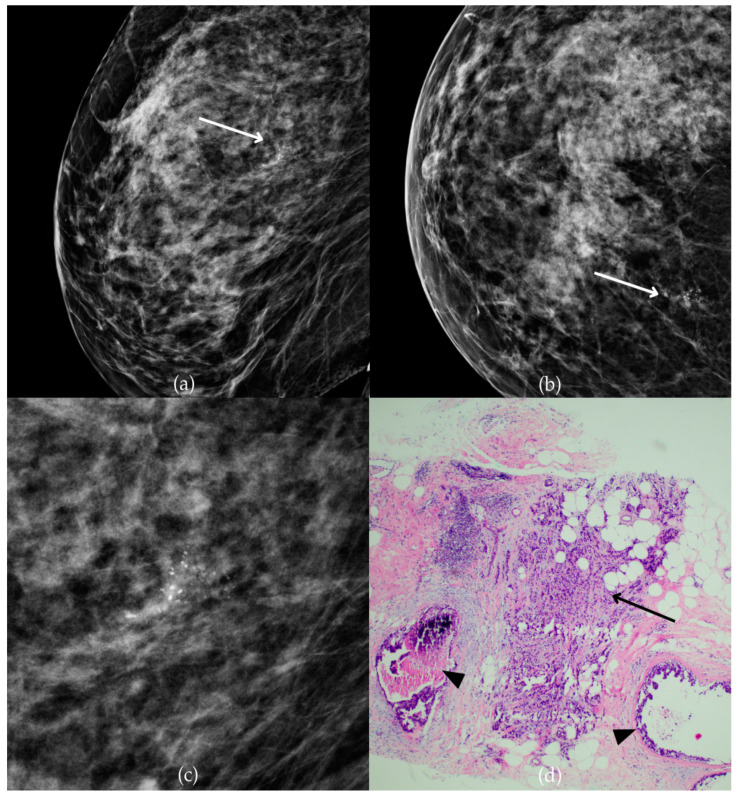
Invasive breast cancer. (**a**) Mediolateral oblique (MLO) mammography view; (**b**) craniocaudal (CC) mammography view of the right breast showing coarse heterogeneous and fine pleomorphic calcifications (marked with arrows) in the upper outer quadrant, with segmental distribution. (**c**) Magnified mammographic image of coarse heterogeneous and fine pleomorphic calcifications with segmental distribution. (**d**) The histopathological image of the biopsy sample shows the characteristic features of invasive cancer (marked with arrow) and ductal carcinoma in situ (DCIS) (marked with arrowhead) (HE staining; 40× magnification).

During a screening program, a 64-year-old patient presented with suspicious microcalcifications. The mammogram shown in Figure 3 of the patient’s right breast revealed a segmental distribution of coarse heterogeneous and fine pleomorphic calcifications in the upper outer quadrant, corresponding to a BI-RADS 4 finding. Tissue analysis obtained through VABB confirmed the presence of invasive carcinoma (category B5b). The patient underwent a mastectomy. Postoperative pathology showed only an in situ component, with no evidence of an invasive component, indicating that the invasive component was removed during the biopsy. One year after diagnosis, the follow-up US was reported as BI-RADS 2 classification, indicating benign imaging features.

**Figure 4:**
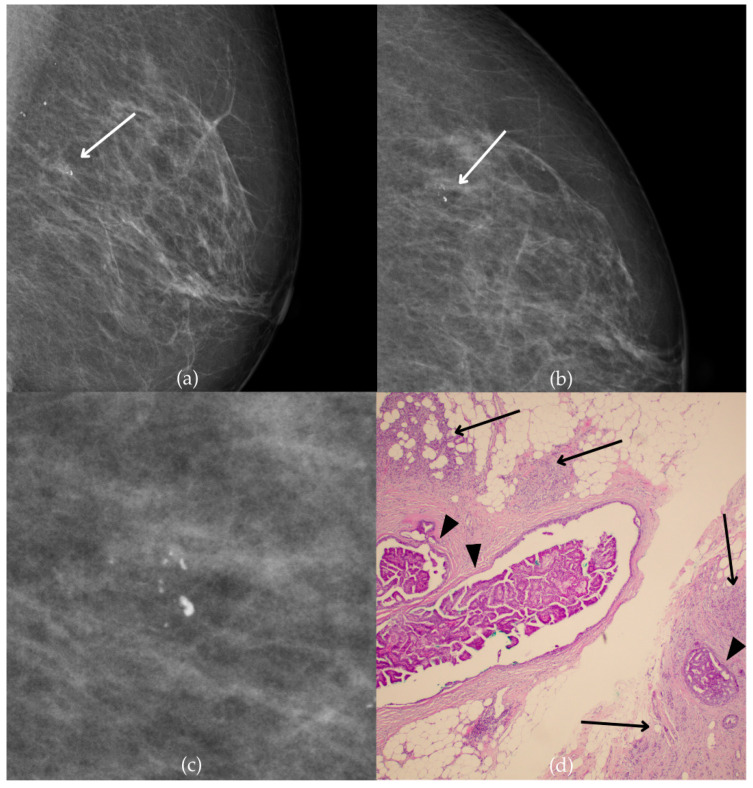
Invasive breast cancer. (**a**) Mediolateral oblique (MLO) mammography view; (**b**) craniocaudal (CC) mammography view of the left breast showing grouped coarse heterogeneous calcifications (marked with arrows) in the upper outer quadrant. (**c**) Magnified mammographic image of grouped coarse heterogeneous calcifications. (**d**) The histopathological image of the biopsy sample shows the characteristic features of invasive cancer (marked with arrow) and ductal carcinoma in situ (DCIS) (marked with arrowhead) (HE staining; 40× magnification).

The mammogram and histopathological images (Figure 4) of the 62-year-old patient’s left breast show grouped coarse heterogeneous calcifications in the upper outer quadrant, corresponding to a BI-RADS 3 finding. Tissue analysis obtained through biopsy indicated the presence of an invasive carcinoma (category B5b). The patient underwent quadrantectomy, and postoperative pathology confirmed the diagnosis of invasive carcinoma. The patient’s most recent MRI, performed two years after the initial diagnosis, was assessed as BI-RADS 2, suggesting benign changes.

**Figure 5:**
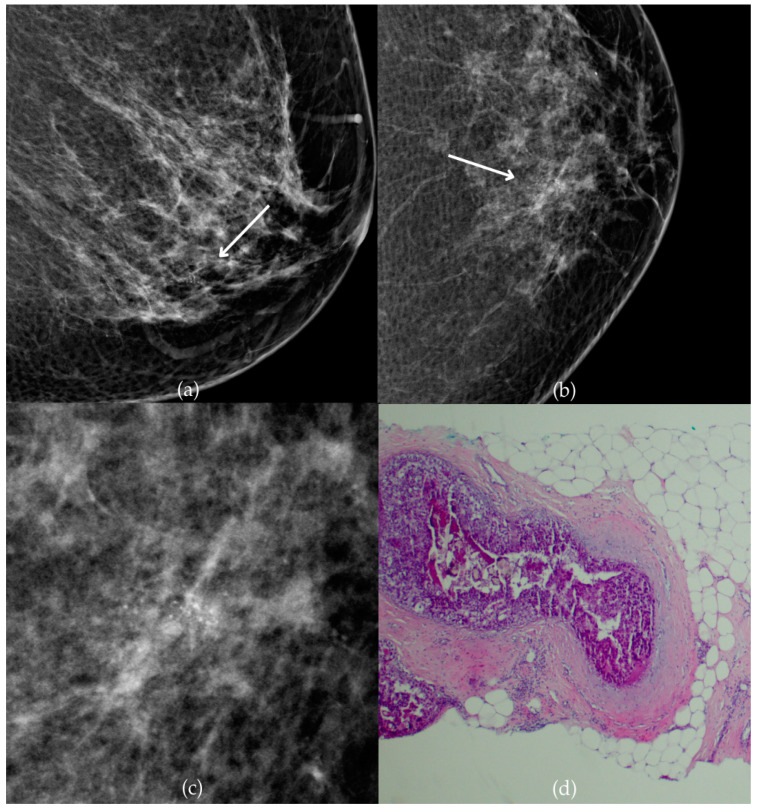
Ductal carcinoma in situ (DCIS). (**a**) Mediolateral oblique (MLO) view; (**b**) craniocaudal (CC) view of the left breast showing architectural distortion with associated fine pleomorphic calcifications (marked with arrows) in the lower inner quadrant, with a linear distribution. (**c**) Magnified mammographic image of fine pleomorphic calcifications with a linear distribution within architectural distortion. (**d**) The histopathological image of the biopsy sample shows the characteristic features of DCIS (HE staining; 40× magnification).

Figure 5 presents mammographic and histopathological images of a 56-year-old patient. The mammogram of the patient’s left breast shows architectural distortion within which there are fine pleomorphic calcifications with linear distribution in the lower inner quadrant, corresponding to a BI-RADS 4 finding. Tissue analysis obtained through biopsy indicated the presence of DCIS (category B5a). The patient underwent a mastectomy, and postoperative pathology detected invasive carcinoma.

**Figure 6:**
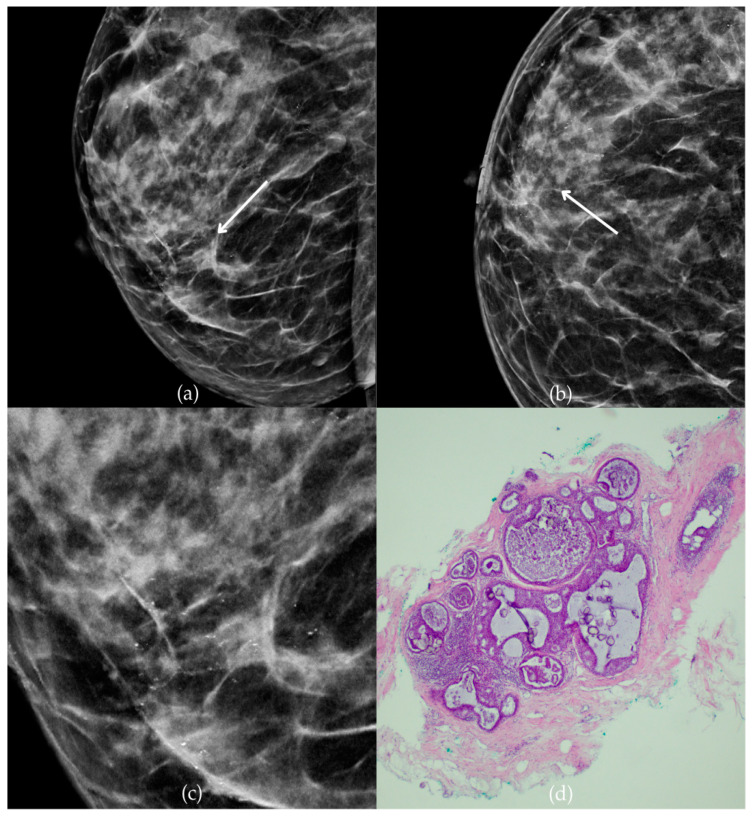
Ductal carcinoma in situ (DCIS). (**a**) Mediolateral oblique (MLO) view; (**b**) craniocaudal (CC) view of the right breast showing amorphous calcifications of regional distribution (marked with arrows) in the outer quadrants border. (**c**) Magnified mammographic image of amorphous calcifications of regional distribution. (**d**) The histopathological image of the biopsy sample shows the characteristic features of DCIS (HE staining; 100× magnification).

Figure 6 presents mammographic and histopathological images of a 49-year-old patient. The mammogram of the patient’s right breast shows amorphous calcifications of regional distribution in the outer quadrants border, corresponding to a BI-RADS 3 finding. Tissue analysis obtained through biopsy confirmed the presence of DCIS (category B5a). The patient underwent a mastectomy, and postoperative pathology confirmed the diagnosis of carcinoma in situ. One year after diagnosis, the follow-up MRI and US were reported as BI-RADS 2, confirming the absence of malignancy.

**Figure 7:**
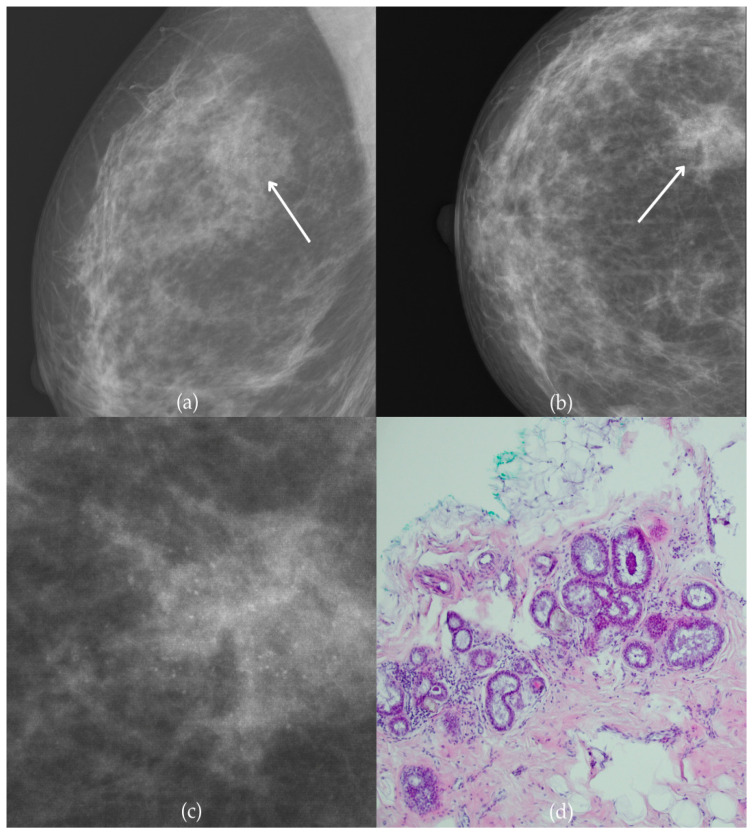
Flat epithelial atypia (FEA). (**a**) Mediolateral oblique (MLO) view; (**b**) craniocaudal (CC) view of the right breast showing amorphous calcifications of regional distribution (marked with arrows) in the upper outer quadrant. Associated parenchyma asymmetry is also noted. (**c**) Magnified mammographic image of amorphous calcifications of regional distribution. (**d**) The histopathological image of the biopsy sample shows FEA (HE staining; 100× magnification).

In Figure 7, mammographic and histopathological images illustrate the findings in a 46-year-old patient. The mammogram of the patient’s right breast shows amorphous calcifications over the 3 cm area (regional distribution) in the upper outer quadrant, corresponding to a BI-RADS 4 finding. Tissue analysis was obtained through biopsy and reported FEA (category B3). The patient underwent an excisional biopsy (quadrantectomy), and postoperative pathology confirmed the absence of carcinoma. At the latest follow-up, conducted six years post-diagnosis, the MRI was categorized as BI-RADS 2, indicating no suspicious findings.

**Figure 8:**
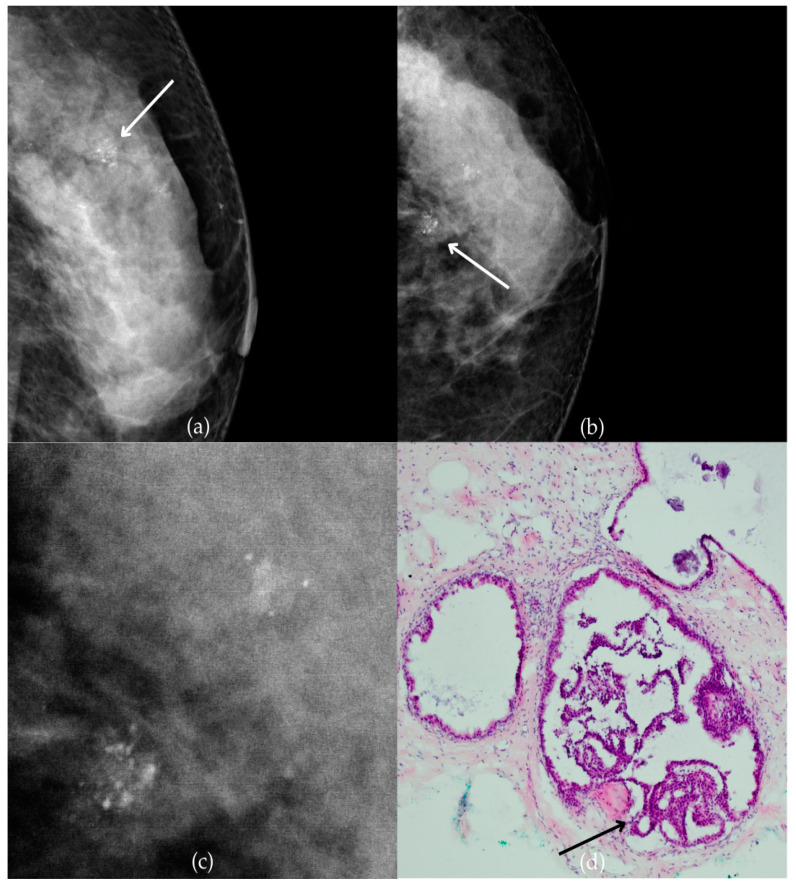
Flat epithelial atypia (FEA) and atypical ductal hyperplasia (ADH). (**a**) Mediolateral oblique (MLO) view; (**b**) craniocaudal (CC) view of the left breast showing architectural distortion within which grouped amorphous and some coarse heterogeneous calcifications (marked with arrows) are seen in the upper outer quadrant. (**c**) Magnified mammographic image of a grouped amorphous and some coarse heterogeneous calcifications. (**d**) The histopathological image of the biopsy sample shows FEA and ADH (marked with arrows) (HE staining; 100× magnification).

In Figure 8, mammographic and histopathological images illustrate the findings in a 51-year-old patient. The mammogram of the patient’s left breast shows architectural distortion with associated grouped amorphous and some coarse heterogeneous calcifications in the upper outer quadrant. Tissue analysis was obtained through biopsy, and both FEA and ADH (category B3) were identified. The patient underwent an excisional biopsy (quadrantectomy), and postoperative pathology confirmed the absence of carcinoma.

**Figure 9:**
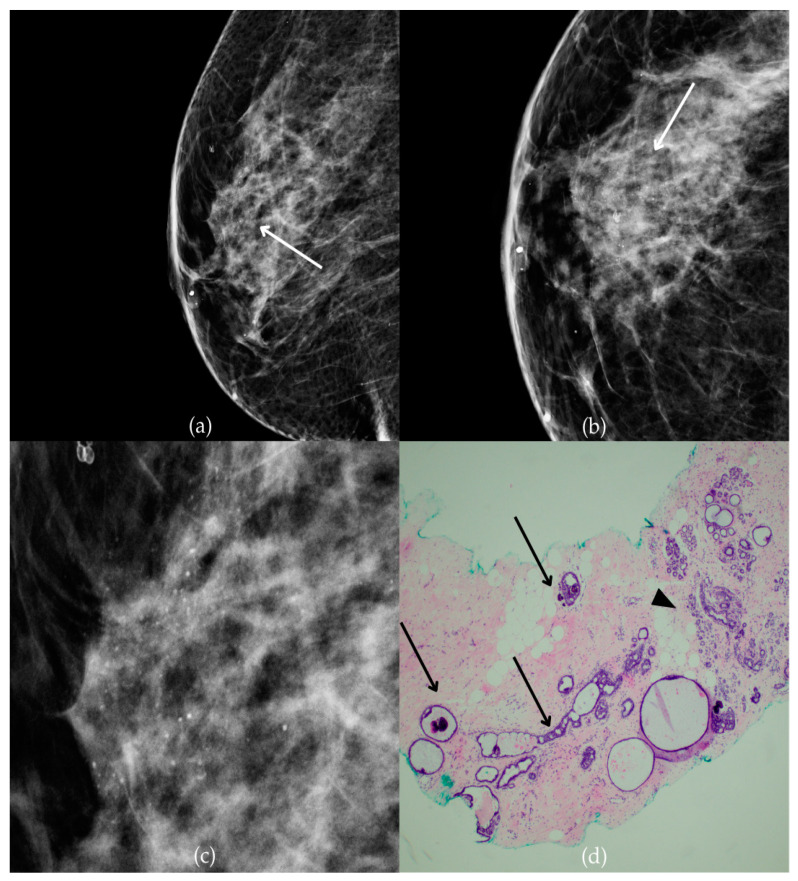
Atypical ductal hyperplasia (ADH) and sclerosing adenosis (SA). (**a**) Mediolateral oblique (MLO) view; (**b**) craniocaudal (CC) view of the right breast showing fine pleomorphic calcifications of regional distribution (marked with arrows) in the upper outer quadrant. (**c**) Magnified mammographic image of fine pleomorphic calcifications of regional distribution. (**d**) The histopathological image of the biopsy sample shows ADH (marked with arrows) and SA (marked with arrowhead) (HE staining; 100× magnification).

Figure 9 presents mammographic and histopathological images of a 54-year-old patient. The mammogram of the patient’s right breast shows fine pleomorphic calcifications of regional distribution in the upper outer quadrant, corresponding to a BI-RADS 4 finding, due to which a biopsy was performed. Tissue analysis reported ADH and sclerosing adenosis (SA) (category B3). The patient underwent an excisional biopsy (quadrantectomy), and postoperative pathology confirmed the absence of carcinoma. At the most recent follow-up, conducted one year after the initial diagnosis, the US examination was classified as BI-RADS 2, indicating benign findings.

**Figure 10:**
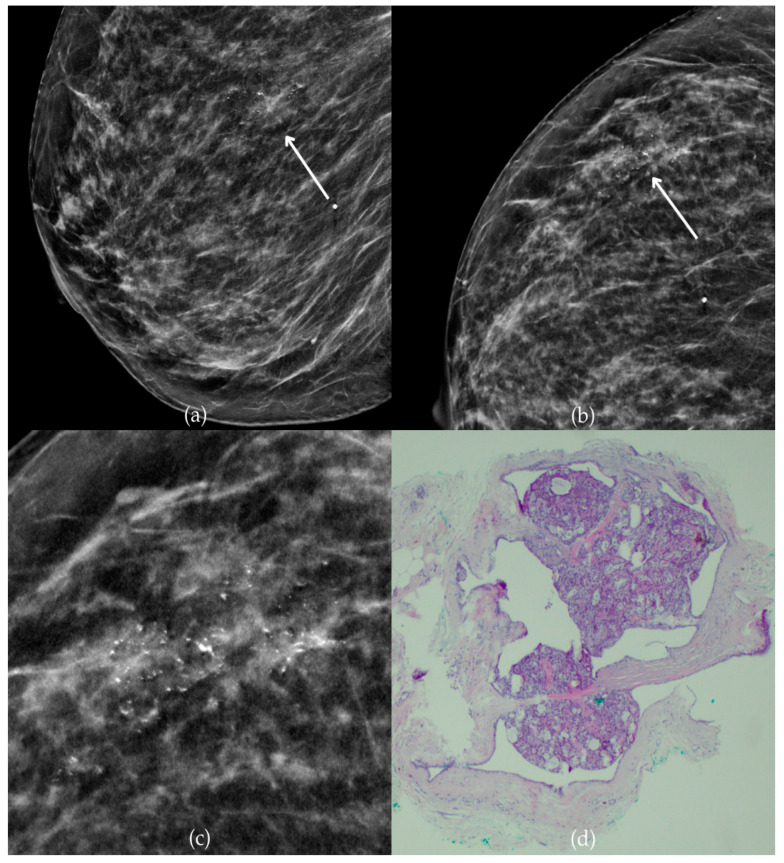
Intraductal papillary lesion without epithelial atypia. (**a**) Mediolateral oblique (MLO) view; (**b**) craniocaudal (CC) view of the right breast showing architectural distortion with associated fine pleomorphic calcifications of segmental distribution (marked with arrows) in the upper outer quadrant. (**c**) Magnified mammographic image of fine pleomorphic calcifications of segmental distribution. (**d**) The histopathological image of the biopsy sample shows an intraductal papillary lesion without epithelial atypia (HE staining; 40× magnification).

Mammogram and histopathological images (Figure 10) of a 66-year-old patient’s right breast show architectural distortion with associated fine pleomorphic calcifications of segmental distribution in the upper outer quadrant, corresponding to a BI-RADS 3 finding. Tissue analysis obtained through biopsy reported an intraductal papillary lesion without epithelial atypia (category B3). The patient underwent an excisional biopsy (quadrantectomy), and postoperative pathology confirmed the absence of carcinoma. One year after the initial diagnosis, the most recent US follow-up was classified as BI-RADS 2, confirming benign findings.

## 6. Discussion and Recommendations

The pictorial review serves as a visual reference, presenting representative cases to assist in the recognition and interpretation of mammographic findings. Table 1 summarizes the characteristics of microcalcifications observed in the presented cases, emphasizing their similarities and the inherent challenge of diagnosing lesions based solely on imaging features.

According to the BI-RADS classification, amorphous, pleomorphic, and coarse heterogeneous calcifications are considered suspicious. All cases presented in this study fall into this category. Linear, segmental, and grouped distributions are generally associated with a higher likelihood of malignancy [7]. However, in Case 6, the calcifications exhibit a regional distribution, which typically suggests a benign process. Yet, in this instance, the findings correspond to DCIS, demonstrating that malignancy can occasionally present with features usually linked to benign conditions. The likelihood of malignancy is generally higher when architectural distortion is present compared to cases where there is no distortion [7]. In our study, architectural distortion was observed in both malignant and benign cases, reinforcing the need for careful assessment. While microcalcifications are a key marker for detecting DCIS and other high-risk lesions, their appearance on imaging alone is insufficient for a definitive diagnosis. This points out the critical role of histopathological confirmation and the necessity of an accurate biopsy method specifically designed for the evaluation of microcalcifications. Considering all the above, stereotactic-guided VABB is particularly effective and recommended in these cases, as it allows for precise sampling of microcalcifications, even when no associated mass is present. Since many of these lesions are not visible on ultrasound, stereotactic guidance provides a reliable targeting system, ensuring an accurate and minimally invasive diagnostic approach. Beyond its diagnostic advantages, VABB plays a crucial role in reducing unnecessary surgical interventions. Given its high diagnostic yield, it should be prioritized over surgical excision whenever feasible to minimize patient discomfort, lower healthcare costs, and prevent overtreatment of benign lesions [23,24]. By adopting standardized protocols, encouraging interdisciplinary collaboration, and continuing research into optimized diagnostic strategies, we can further improve breast cancer detection and patient care.

## 7. Conclusions

This review emphasizes the role of VABB in breast imaging, especially in cases of microcalcifications found by mammography. Breast cancer is the leading cause of morbidity and mortality in women; therefore, early detection and precise diagnosis are essential. Microcalcifications are often benign but are the earliest sign of DCIS and invasive malignancies and require accurate evaluation to achieve the best patient outcome. VABB is a minimally invasive procedure that gives adequate tissue samples and a more reliable diagnosis. Stereotactic guidance makes it even more precise. The ability to minimize surgical excision and allow comprehensive histopathological analysis enables better pre-operative planning and less intervention. By adding pictorial reviews and real case scenarios, practitioners can gain a better understanding of various breast pathologies and imaging findings. A combination of theory and practical application will improve patient outcomes. As breast imaging evolves, VABB and case-based learning will be part of the modern diagnostic approach. The limitations of VABB include its unsuitability for certain lesions and limited accessibility in some clinical settings. While VABB offers high diagnostic accuracy, its long-term effectiveness still requires further investigation through large-scale studies to fully understand its role in patient management and long-term clinical outcomes.

## 8. Future Direction

Future research on VABB should focus on enhancing its diagnostic accuracy by integrating advanced imaging technologies. Contrast-enhanced mammography (CEM)-guided biopsy has emerged as a promising alternative to MRI-guided biopsy for the evaluation of enhancing-only breast lesions. With a reported success rate of over 95% and a median procedure time of 15 min, CEM-guided biopsy offers a more accessible and cost-effective option, particularly in centers where MRI is not available [33]. The integration of artificial intelligence in lesion detection and procedural planning may also contribute to greater precision and efficiency. Recent progress in AI applications for mammography has shown promising results in lesion detection, classification, and risk assessment. Deep learning models have demonstrated the ability to match radiologists’ performance in identifying suspicious findings. AI algorithms are being increasingly integrated into clinical workflows to assist radiologists in reducing false positives, improving lesion localization, and prioritizing high-risk cases for further evaluation. Further research is needed to ensure that AI models trained on specific datasets can be effectively applied across diverse populations and imaging systems. Additionally, future studies should explore the role of AI in predicting tumor aggressiveness, response to therapy, and long-term patient outcomes based on mammographic patterns [34,35].

## Figures and Tables

**Table 1:** Overview of microcalcification characteristics.

Case Number	Calcification Morphology	Distribution of Calcification	Architectural Distortion	Diagnosis
1	Coarse heterogeneous and fine pleomorphic	Grouped	No	DCIS
2	Amorphous	Grouped	Yes	DCIS
3	Coarse heterogeneous and fine pleomorphic	Segmental	No	Invasive breast cancer
4	Coarse heterogeneous	Grouped	No	Invasive breast cancer
5	Fine pleomorphic	Linear	Yes	DCIS
6	Amorphous	Regional	No	DCIS
7	Amorphous	Regional	No	FEA
8	Amorphous and coarse heterogeneous	Grouped	Yes	FEA, ADH
9	Fine pleomorphic	Regional	No	ADH, SA
10	Fine pleomorphic	Segmental	Yes	Intraductal papillary lesion

DCIS—ductal carcinoma in situ, FEA—flat epithelial atypia, ADH—atypical ductal hyperplasia, SA—sclerosing adenosis.

## Data Availability

All data generated or analyzed during this study are included in this published article.

## Abbreviations

The following abbreviations are used in this manuscript:

US	Ultrasound
MRI	Magnetic resonance imaging
DCIS	Ductal carcinoma in situ
BI-RADS	The Breast Imaging Reporting and Data System
ACR	American College of Radiology
FNAC	Fine-needle aspiration cytology
CNB	Core needle biopsy
VABB	Vacuum-assisted breast biopsy
ADH	Atypical ductal hyperplasia
FEA	Flat epithelial atypia
LN	Classical lobular neoplasia
RS	Radial scar
PL	Papillary lesions
PT	Phyllodes tumors
SA	Sclerosing adenosis
CEM	Contrast-enhanced mammography
